# Mechanoregulation of Osteoclastogenesis-Inducing Potentials of Fibrosarcoma Cell Line by Substrate Stiffness

**DOI:** 10.3390/ijms24108959

**Published:** 2023-05-18

**Authors:** Watcharaphol Tiskratok, Masahiro Yamada, Jun Watanabe, Qu Pengyu, Tsuyoshi Kimura, Hiroshi Egusa

**Affiliations:** 1 Division of Molecular and Regenerative Prosthodontics, Tohoku University Graduate School of Dentistry, 4-1 Seiryo-machi, Sendai 980-8575, Miyagi, Japan; watcharaphol@sut.ac.th (W.T.); jun.watanabe.b4@tohoku.ac.jp (J.W.); qu.pengyu.q7@dc.tohoku.ac.jp (Q.P.); 2 School of Geriatric Oral Health, Institute of Dentistry, Suranaree University of Technology, 111 University Rd. Suranaree, Nakhon Ratchasima 30000, Mueang, Thailand; 3 Department of Material-Based Medical Engineering, Institute of Biomaterials and Bioengineering, Tokyo Medical and Dental University, Tokyo 101-0062, Chiyoda-ku, Japan; kimurat.mbme@tmd.ac.jp; 4 Center for Advanced Stem Cell and Regenerative Research, Tohoku University Graduate School of Dentistry, 4-1 Seiryo-machi, Sendai 980-8575, Miyagi, Japan

**Keywords:** cellular mechanotransduction, extracellular matrix, micro-physiological system, osteoclastogenesis, proinflammatory mediator

## Abstract

A micro-physiological system is generally fabricated using soft materials, such as polydimethylsiloxane silicone (PDMS), and seeks an inflammatory osteolysis model for osteoimmunological research as one of the development needs. Microenvironmental stiffness regulates various cellular functions via mechanotransduction. Controlling culture substrate stiffness may help spatially coordinate the supply of osteoclastogenesis-inducing factors from immortalized cell lines, such as mouse fibrosarcoma L929 cells, within the system. Herein, we aimed to determine the effects of substrate stiffness on the osteoclastogenesis-inducing potential of L929 cells via cellular mechanotransduction. L929 cells showed increased expression of osteoclastogenesis-inducing factors when cultured on type I collagen-coated PDMS substrates with soft stiffness, approximating that of soft tissue sarcomas, regardless of the addition of lipopolysaccharide to augment proinflammatory reactions. Supernatants of L929 cells cultured on soft PDMS substrates promoted osteoclast differentiation of the mouse osteoclast precursor RAW 264.7 by stimulating the expression of osteoclastogenesis-related gene markers and tartrate-resistant acid phosphatase activity. The soft PDMS substrate inhibited the nuclear translocation of YES-associated proteins in L929 cells without reducing cell attachment. However, the hard PDMS substrate hardly affected the cellular response of the L929 cells. Our results showed that PDMS substrate stiffness tuned the osteoclastogenesis-inducing potential of L929 cells via cellular mechanotransduction.

## 1. Introduction

Microphysiological systems are innovative preclinical test tools that simulate the response of living human tissues and cells in vitro to improve the efficiency of drug discovery, develop disease models, and establish personalized medicine [1]. In particular, a system that replicates complex immune reactions has garnered attention for overcoming a wide variety of diseases, including inflammatory osteolysis [2]. The establishment of microphysiological systems requires the spatial and temporal construction of a specific microenvironment that controls the function of target cells [3]. A microphysiological system generally consists of multiple segments, including a chamber to supply a specific substance to the target cells. Microenvironments that deliver mechanical stress, electrical or optical signals, or biochemical cues are used in the interior design of each segment [4]. The interior design of each segment should be as simple as possible so that the system can be adapted for high-throughput studies [5]. Static mechanical stress from a substrate in the chamber using topographic and physical cues is favorable for spatial-specific cellular control within the system [6].

Osteoclastogenesis is a critical osteoimmunological event involved in the pathogenesis of degenerative bone diseases [7]. In vitro osteoclastogenesis needs to control the differentiation of macrophages and preosteoclastic cells by co-culturing with a cocktail of various proinflammatory mediators, such as prostaglandin E2 (PGE_2_), macrophage colony-stimulating factor (M-CSF), tumor necrosis factor-alpha (TNF-α) and receptor activator of nuclear factor-kappa B ligand (RANKL) [7,8]. The application of exogenous reagents is a technological issue in microfluidic devices owing to complicated mixing procedures, such as dilution, pre-reaction control, and sustained supply [9]. One of the approaches for osteoclastogenesis within the device is to incorporate immortalized cell lines that produce proinflammatory mediators into the system, such as the fibrosarcoma-derived fibroblast cell line L929 [10]. These cell lines have been successfully incorporated into various types of microfluidic devices [11,12]. Furthermore, the culture supernatant of L929 cells can promote proinflammatory responses of cultured macrophages more than artificially added M-CSF [13] because the supernatant is a cytokine cocktail containing not only M-CSF to drive macrophage differentiation but also other factors that regulate macrophage phenotypes [14]. Therefore, spatial-specific controls in the production of osteoclastogenesis-inducing factors from immortalized cell lines are useful for designing an inner microenvironment to assess osteoclastogenesis in microphysiological systems.

Cells activate mechanotransduction by sensing mechanical signals from their surrounding environment [15]. Fibroblasts are representative cells that alter inflammation and matrix production and degradation based on mechanical signals from the surrounding environment. For instance, oral mucosal fibroblasts may activate immune cells toward bone resorption by producing proinflammatory cytokines and mediators in response to cyclic pressure loading [16]. The biomimetic physical microenvironment of the artificial tooth surface can induce *in situ* periodontal tissue regeneration by tuning periodontal ligament fibroblastic cells [17]. Stiffness of the microenvironment as static mechanical stress potentially tunes cellular pro-inflammatory responses via yes-associated protein (YAP)-mediated mechanotransduction [18] and is involved in various inflammatory diseases related to fibroblasts, such as tissue fibrosis and tumor progression [19,20,21]. Fibroblasts cultured on stiffer matrices increased collagen production by reducing PGE_2_ and matrix metalloprotease (MMP) production, whereas they increased PGE_2_ and MMP production on softer matrices [22]. Stiffness-mediated proinflammatory microenvironments can be produced using polydimethylsiloxane silicone (PDMS) substrates, which are representative base materials for microphysiological systems. Soft PDMS substrates induce the expression of proinflammatory mediators and impair collagen synthesis in human gingival fibroblasts through the suppression of YAP nuclear translocation, even without ligand stimulation, whereas hard substrates induce opposite reactions [23]. A stiffer photoresist substrate changes the morphology of L929 cells from polygonal to spindle shapes [24]; this suggests the generation of an intrinsic force within the cell body that affects YAP-mediated mechanotransduction [25].

Based on this background, we hypothesized that soft substrates increase the production of osteoclastogenesis-inducing factors in immortalized cell lines by inhibiting cellular mechanotransduction, whereas hard substrates induce opposite reactions. In this study, we aimed to determine whether the stiffness of PDMS-based substrates regulates the osteoclastogenesis-inducing potential of L929 cells via YAP-mediated mechanotransduction.

## 2. Results

### 2.1. Determination of Culture Conditions

#### 2.1.1. Collagen Coating Conditions on the PDMS Substrate

**Figure 1:**
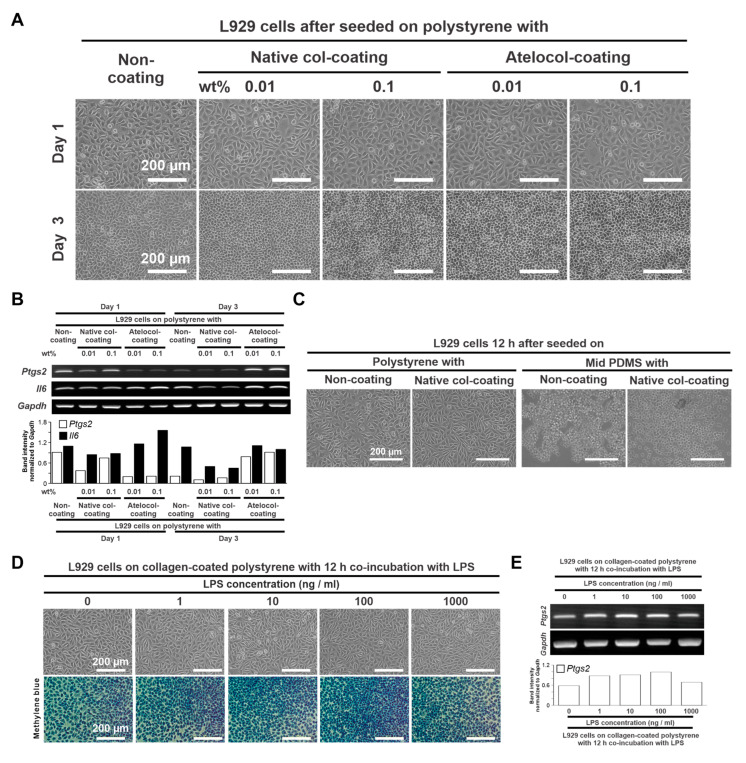
Determination of the conditions of the PDMS-based culture substrate. (**A**) Representative microscopic images of L929 cells cultured on polystyrene culture plates coated with 0.01 wt% and 0.1 wt% native collagen or atelocollagen at days 1 (upper images) and 3 (lower images). (**B**) Gene expression of *Ptgs2*, *Il6*, and *Gapdh* was analyzed by agarose gel electrophoresis-based RT–PCR in L929 cells under the corresponding culture conditions as above, the histogram of *Ptgs2* and *Il6* band intensities normalized to that of *Gapdh*. (**C**) Representative microscopic images of L929 cells cultured on a polystyrene culture plate and the mid PDMS substrate with and without native collagen coating for 12 h. (**D**) Representative images of L929 cells cultured on the 0.01 wt% collagen-coated polystyrene and co-incubated with 0, 10, 100, and 1000 ng/mL of LPS for 12 h under a phase microscope (upper) and a light microscope (lower) after methylene blue staining. (**E**) Gene expression of *Ptgs2* and *Gapdh* was analyzed by agarose gel electrophoresis-based RT–PCR in L929 cells under the corresponding culture conditions as above; the histogram of *Ptgs2* band intensity normalized to that of *Gapdh*. PDMS, Polydimethylsiloxane; RT–PCR, Reverse transcription–polymerase chain reaction; Col, Collagen; Ptgs2, Prostaglandin G/H synthase 2; Il6, Interleukin-6; Gapdh, Glyceraldehyde-3-phosphate dehydrogenase; LPS, Lipopolysaccharide; SD, Standard deviation; HSD, Honestly significant difference.

PDMS substrates with 4.4 kPa, 17 kPa, or 26.2 kPa in Young’s modulus were prepared as soft, mid, or hard PDMS substrates, respectively, according to previously reported methods [23]. To optimize the type I collagen coating on the substrates for cell attachment, the L929 cells were cultured on a culture-graded polystyrene plate or the mid PDMS, which were coated with 0.01 wt% or 0.1 wt% native collagen or atelocollagen. On days 1 and 3, both the native collagen- and atelocollagen-coating were similar in appearance compared with that of the L929 cell culture on the non-coated polystyrene, regardless of the concentration (Figure 1A). In addition, the expression of proinflammatory gene markers, such as prostaglandin-endoperoxide synthase 2 (*Ptgs2*) and interleukin 6 (*Il6*), was consistently low at both concentrations of native collagen-coated polystyrene on days 1 and 3 (Figure 1B). Atelocollagen-coated polystyrene was apparently higher in these gene expressions than native one, except for less expression of the *Ptgs2* gene at day 1 (Figure 1B). The 0.01 wt% native collagen coating allowed L929 cells to attach to the mid-PDMS 12 h after seeding, in contrast to the inhibited cellular attachment on non-coated PDMS (Figure 1C). Therefore, a 0.01 wt% native collagen coating was used for subsequent experiments.

#### 2.1.2. Concentration of an Inflammatory Ligand

Lipopolysaccharide (LPS) has been used to augment the proinflammatory responses of fibroblastic cells by stimulating the proinflammatory ligand toll-like receptor 4 [26]. To determine the LPS concentration tested, L929 cells were cultured on collagen-coated polystyrene with or without co-incubation with LPS for 12 h. Both phase microscope images of the cell culture and light microscope images of the corresponding culture with methylene blue staining showed that the culture co-incubated with 1000 ng/mL LPS looked like the culture without LPS in terms of cell density, in contrast with the denser cell density in the culture co-incubated with the other LPS concentrations (Figure 1D). *Ptgs2* gene expression in the culture 12 h after seeding was upregulated by co-incubating with LPS, regardless of the concentration (Figure 1E). Therefore, 1000 ng/mL LPS was used for subsequent L929 culture experiments.

### 2.2. Effects of Substrate Stiffness on the Production of Proinflammatory Mediators of L929 Cells

**Figure 2:**
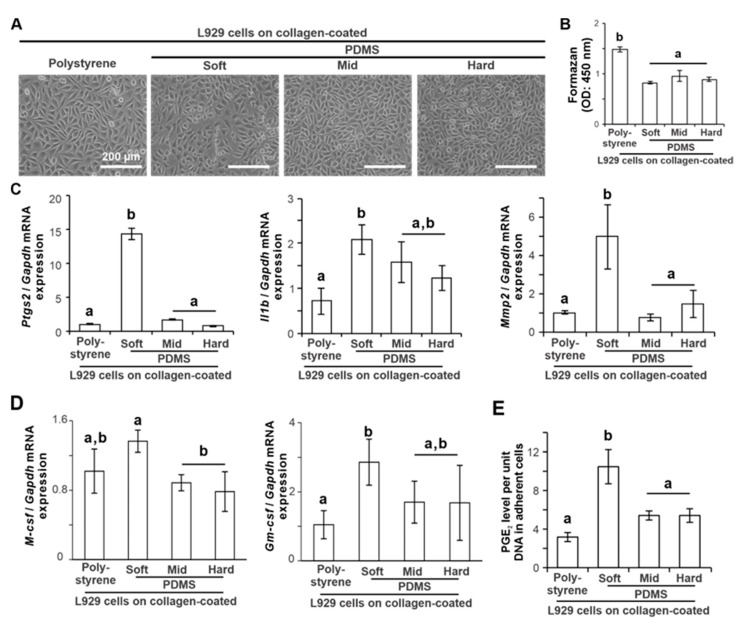
Effects of substrate stiffness on the production of proinflammatory mediators of L929 cells. (**A**) Representative microscopic image of L929 cells cultured on a 0.01 wt% collagen-coated polystyrene culture plate and soft, mid, and hard PDMS for 12 h. Results of the amounts of adherent cells measured with WST-1 colorimetry (**B**) and gene expression of *Ptgs2*, *Il1b*, *Mmp2* (**C**), *M-csf*, and *Gm-csf* (**D**) relative to *Gapdh* analyzed by RT–PCR in L929 cell cultures under the corresponding culture conditions described above. (**E**) PGE_2_ level per unit DNA in adherent L929 cells under the corresponding culture conditions as above using ELISA on culture supernatants. Data are represented as means ± standard deviation (SD) (*n* = 3). Different lowercase letters in histograms indicate statistically significant differences between them (*p* < 0.05; Tukey’s HSD). PDMS, Polydimethylsiloxane; OD, Optical density; Ptgs2, Prostaglandin G/H synthase 2; Il1b, Interleukin-1β; Mmp2, Matrix metallopeptidase-2; M-csf, Macrophage colony-stimulating factor; Gm-csf, Granulocyte-macrophage colony-stimulating factor; Gapdh, Glyceraldehyde-3-phosphate dehydrogenase; PGE_2_, Prostaglandin E2; RT–PCR, reverse transcriptase–polymerase chain reaction; HSD, honestly significant different; ELISA, enzyme-linked immunosorbent assay.

L929 cells cultured on a collagen-coated polystyrene culture plate and soft, mid, and hard PDMS showed that the adherent cell densities were low on soft PDMS and increased with increasing PDMS stiffness (Figure 2A). The number of adherent L929 cells on the PDMS substrates 12 h after seeding was lower than that on polystyrene (Figure 2B) (*p* < 0.05, Tukey’s HSD test) but did not differ among the types of PDMS substrate (*p* > 0.05, Tukey’s HSD test). The expression of proinflammatory and extracellular matrix (ECM) destruction gene markers for *Ptgs2*, *Il1b*, and *Mmp2* in L929 cells 12 h after seeding was consistently upregulated on soft PDMS compared to polystyrene (Figure 2C) (*p* < 0.05, Tukey’s HSD test), in contrast with no difference in those gene levels on the other PDMSs (*p* > 0.05, Tukey’s HSD test). The expression of *M-csf* was higher on soft PDMS than on the other PDMSs (Figure 2D) (*p* < 0.05, Tukey’s HSD test), whereas the expression of the granulocyte-macrophage colony-stimulating factor (*Gm-csf*) gene on soft PDMS was higher than that on polystyrene (*p* < 0.05, Tukey’s HSD test), but was not significantly different from that on the other PDMSs (*p* > 0.05, Tukey’s HSD test). The PGE_2_ levels per unit DNA in adherent L929 cells determined by enzyme-linked immunosorbent assay (ELISA) of the culture supernatants were 2–3 times higher on soft PDMS than on polystyrene and other PDMSs (Figure 2E) (*p* < 0.05, Tukey’s HSD test).

### 2.3. Effects of Substrate Stiffness on the Production of Proinflammatory Mediators of L929 Cells under Additional Ligand Stimulation

**Figure 3:**
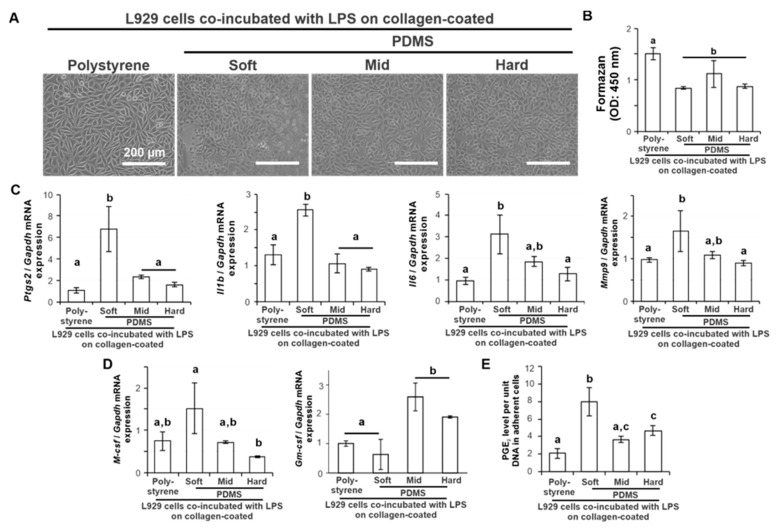
Effects of substrate stiffness on the production of proinflammatory mediators of L929 cells under ligand stimulation. Representative phase microscopic images (**A**), the amounts of adherent cells measured with WST-1 colorimetry (**B**), and RT–PCR-analyzed gene expression of *Ptgs2*, *Il1b*, *Il6*, *Mmp9* (**C**), *M-csf*, and *Gm-csf* (**D**) relative to *Gapdh* in L929 cells cultured on the 0.01 wt% collagen-coated polystyrene culture plate and soft, mid, and hard PDMS, co-incubated with 1000 ng/mL LPS for 12 h. (**E**) PGE_2_ level per unit DNA in adherent L929 cells on the 0.01 wt% collagen-coated polystyrene culture plate and soft, mid, and hard PDMS, co-incubated with 1000 ng/mL LPS for 12 h using ELISA on culture supernatants. Data are represented as means ± standard deviation (SD) (*n* = 3). Different lowercase letters in the histograms indicate statistically significant differences (*p* < 0.05, Tukey’s HSD). LPS, Lipopolysaccharide; PDMS, Polydimethylsiloxane; OD, Optical density; Ptgs2, Prostaglandin G/H synthase 2; Il1b, Interleukin-1β; Il6, Interleukin-6; Mmp9, Matrix Metallopeptidase-9; M-csf, Macrophage colony stimulating factor; Gm-csf, Granulocyte macrophage colony-stimulating factor; Gapdh, Glyceraldehyde-3-phosphate dehydrogenase; PGE_2_, Prostaglandin E2; RT–PCR, reverse transcriptase-polymerase chain reaction; HSD, honestly significant different; ELISA, enzyme-linked immunosorbent assay.

The adherent L929 cells after co-incubation with LPS for 12 h looked apparently sparse on the soft PDMS compared to those on the polystyrene or other PDMSs (Figure 3A). However, the number of adherent L929 cells on the PDMS substrate 12 h after seeding did not differ among the different types of PDMS substrates (Figure 3B) (*p* > 0.05, Tukey’s HSD test). The expression of proinflammatory and tissue destruction gene markers for *Ptgs2*, *Il1b*, *Il6*, and *Mmp9* in L929 cells 12 h after seeding was consistently upregulated on soft PDMS compared to polystyrene (Figure 3C) (*p* < 0.05, Tukey’s HSD test), in contrast with no difference in the gene levels on the other PDMSs (*p* > 0.05, Tukey’s HSD test). The expression of *M-csf* after LPS co-incubation for 12 h was upregulated on soft PDMS more than on hard PDMS (Figure 3D) (*p* < 0.05, Tukey’s HSD test), which did not differ in the gene expression level from polystyrene and mid-PDMS (*p* > 0.05, Tukey’s HSD test). In contrast, the expression of *Gm-csf* was higher on the mid and hard PDMS than on the soft PDMS (Figure 3D) (*p* < 0.05, Tukey’s HSD test), which was not different in expression from polystyrene (*p* > 0.05, Tukey’s HSD test). The PGE_2_ levels per unit DNA in adherent cells were 2–4 times higher on soft PDMS than on polystyrene and other PDMSs (Figure 3E) (*p* < 0.05, Tukey’s HSD test).

### 2.4. Effects of Substrate Stiffness on the Production of Osteoclastogenesis-Inducing Factor from L929 Cells

**Figure 4:**
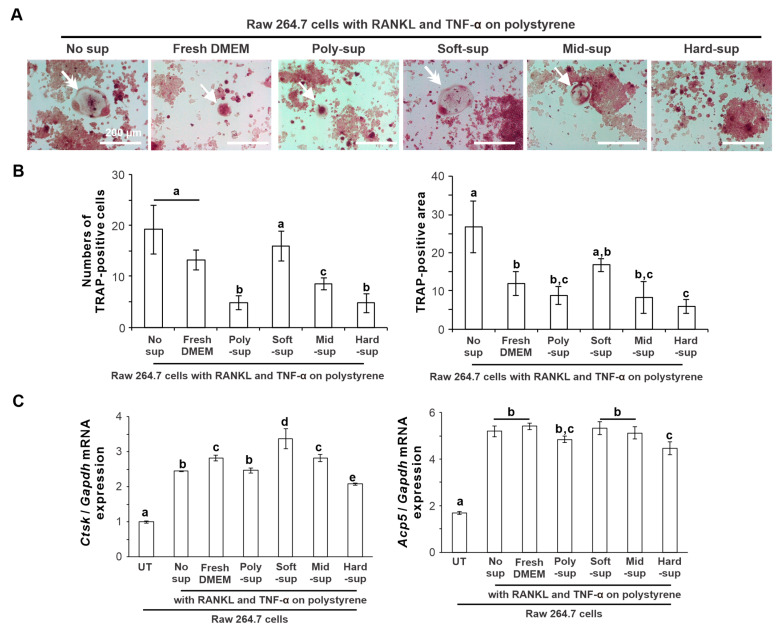
Effects of substrate stiffness on the production of osteoclastogenesis-inducing factors in L929 cells. (**A**) Representative microscopic images of TRAP-stained RAW264.7 cells co-cultured with 50 ng/mL of recombinant human soluble RANKL and 50 ng/mL of TNF-α for 5 days on polystyrene culture plates in Minimum Essential Medium, Alpha modification mixed with or without fresh DMEM, or a DMEM supernatant from L929 cells cultured on the 0.01 wt% collagen-coated polystyrene culture plate and soft, mid, and hard PDMS for 12 h. (**B**) The number of TRAP-positive cells and TRAP-positive area was measured on microscopic images of TRAP-stained cultures using Image J software version 1.53t. (**C**) Gene expression of Ctsk and Acp5 relative to Gapdh was analyzed using RT–PCR in RAW264.7 cells under culture conditions without any osteoclastogenesis-inducing reagents or the corresponding culture conditions mentioned above. Data are represented as means ± standard deviation (SD) (*n* = 4 in (**B**), *n* = 3 in (**C**)). Different lowercase letters in the histograms indicate statistically significant differences between them (*p* < 0.05, Tukey’s HSD test). No-sup, without an L929 cell culture supernatant; Poly-, Soft-, Mid-, or Hard-sup, a supernatant from L929 culture on corresponding surfaces of polystyrene or soft, mid, or hard PDMS; RANKL, receptor activator of nuclear factor-kappa B ligand; TNF-α, tumor necrosis factor-alpha; DMEM, Dulbecco’s Modified Eagle’s Medium; TRAP, tartrate-resistant acid phosphatase; PDMS, Polydimethylsiloxane; Ctsk, cathepsin K; Acp5, tartrate-resistant acid phosphatase 5; Gapdh, Glyceraldehyde-3-phosphate dehydrogenase; PDMS, Polydimethylsiloxane; RT–PCR, reverse transcriptase-polymerase chain reaction; HSD, honestly significant different. White arrows on microscopic images of TRAP-stained culture (**A**) indicate the formation of multinuclear large cells, whereas double arrows indicate smaller multinuclear cells.

Murine preosteoclast cell line RAW264.7 cells were co-cultured on a culture-graded polystyrene plate for 5 days in the conditioned medium containing RANKL, TNF-α, and the culture supernatant of L929 cells on the polystyrene or the PDMS substrates. Tartrate-resistant acid phosphatase (TRAP) staining of osteoclasts showed that TRAP-positive multinuclear large cells were observed in the culture co-incubated with the supernatant of L929 cells on soft PDMS or without the supernatant (Figure 4A, arrows). In contrast, the culture co-incubated with fresh basal medium or L929 cell supernatant from the other substrates showed small TRAP-positive cells (Figure 4A, double arrows). The number of TRAP-positive cells was higher in the culture co-incubated with or without the L929 cell supernatant from the soft PDMS or fresh basal medium than in the culture with the supernatant from the other substrates (Figure 4B) (*p* < 0.05, Tukey’s HSD test). TRAP-positive area in the culture without the supernatants was the highest and 2.5 times higher than that in the culture co-incubated with the L929 supernatants from any substrates (*p* < 0.05, Tukey’s HSD test), except for the soft PDMS showing no significant differences (*p* > 0.05, Tukey’s HSD test). Expression of osteoclast differentiation-related gene markers, such as cathepsin K (*Ctsk*) and tartrate-resistant acid phosphatase type 5 (*Acp5*), was upregulated by the addition of RANKL and TNF-α (Figure 4C) (*p* < 0.05, Tukey’s HSD test), and the supernatant of L929 cells on soft PDMS promoted the expression of these genes at the highest level, rivaling or surpassing those of other culture conditions.

### 2.5. Effects of Substrate Stiffness on Cellular Mechanotransduction in L929 Cells

**Figure 5:**
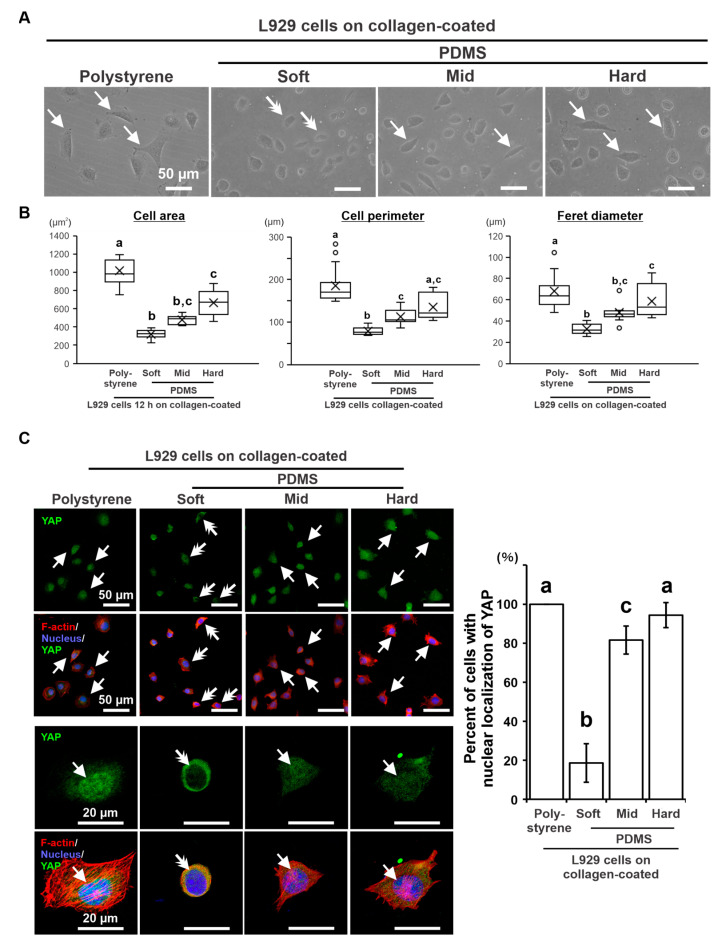
Effects of substrate stiffness on cellular mechanotransduction in L929 cells. Representative phase-contrast microscopic images (**A**) and the corresponding cytomorphometry parameters regarding area, perimeter, and Feret diameter (**B**) in L929 cells cultured on the 0.01 wt% collagen-coated polystyrene culture plate and soft, mid, and hard PDMS for 12 h. (**C**) Low- and high-magnification immunofluorescence confocal microscopy images of YAP (green), F-actin (red), and nucleus (blue) in L929 cells cultured under the conditions indicated. Percentage of cells with YAP in the nucleus (Right). Microscopic images (**A**) showing small and round shapes (double arrows) of L929 cells cultured on soft PDMS, in contrast with spread or spindle shapes (arrows) on polystyrene and other PDMS substrates. Immunofluorescence confocal images (**C**) showing YAP signals in the cytoplasm but not in the nucleus (double arrows) on L929 cells cultured on soft PDMS, in contrast with the signals localized in the nucleus (arrows) on polystyrene. Data are represented as quartiles with a mean (*n* = 15 in (**B**)) or means ± standard deviation (SD) (*n* = 4 in (**C**)). Different lowercase letters in the histograms and boxplot graphs indicate statistically significant differences (*p* < 0.05, Duncan–Bonferroni test or Tukey’s HSD test). PDMS, Polydimethylsiloxane; YAP; Yes-associated protein. White arrows on phase-contrast (**A**) and confocal laser (**C**) microscopic images of the culture indicate elongated cell shapes with nuclear translocation of YAP, whereas double white arrows indicate smaller cells with accumulation of YAP in the cytoplasm but not in the nucleus.

The L929 cells cultured on soft PDMS for 12 h showed relatively small and round shapes, in contrast to the spread or spindle shapes on polystyrene and other PDMS substrates (Figure 5A, arrows). Cytomorphometry showed that the area, perimeter, and Feret diameter 12 h after seeding were the lowest on soft PDMS (Figure 5B) (*p* < 0.05, Dann–Bonferroni test) and tended to increase with increasing PDMS stiffness. The hard PDMS was smaller in area and Feret diameter but not different in perimeter from polystyrene (*p* > 0.05, Dann–Bonferroni test). Immunofluorescence staining showed that YAP was localized in the nuclei of many L929 cells on collagen-coated mid and hard PDMS, although the signals were slightly weaker than those on polystyrene (Figure 5C, arrows). In contrast, YAP was detected only in the cytoplasm of most cells cultured on soft PDMS (Figure 5C, double arrows). The percentage of cells with nuclear YAP increased in a substrate stiffness-dependent manner, being higher in harder substrate (*p* < 0.05, Tukey’s HSD test) and reaching a similar YAP localization profile as that obtained in polystyrene culture plates (Figure 5C).

## 3. Discussion

PDMS has been widely used as a culture substrate that mimics the elastic modulus of living tissues [23,27,28]. Furthermore, their material properties, such as stiffness, optical transparency, low water permeability, and nonconductivity, are favorable for the fabrication of microphysiological systems [29]. However, its hydrophobicity results in low cell-attachment capabilities [30] as shown in Figure 1C. Many surface modification techniques can be used for PDMS to improve its cell-attachment capabilities [29]. In this study, PDMS surfaces were coated with native type I collagen containing a cell adhesion domain for fibroblasts [31]. This type of collagen retains C- and N-terminal telopeptides with antigenic potential [32] but was more cytocompatible with L929 cells than atelocollagen in this study. The high biocompatibility and cytocompatibility of this native collagen were also confirmed in previous in vivo and in vitro studies using the same material as the vehicle or coating material [17,23]. Collagen proteins are among the most highly absorbable extracellular matrices on PDMS surfaces [33] and increase the hydrophilicity of the PDMS surface [34]. In this study, the improvement of L929 cell attachment indicated a successful type I collagen coating on the PDMS substrates by simply covering the PDMS surface with a 0.01% acidic collagen solution. In addition, the native type I collagen coating is resistant to the long-term culture of fibroblastic cells [23] and may be relatively stable to shear stress [29]. Furthermore, LPS was used as a ligand to stimulate the proinflammatory receptors of L929 cells in this study, as in reports of the development of microphysiological systems using other types of cells [35]. The concentration of LPS successfully augmented the production of proinflammatory mediators in L929 cells without inhibiting cell attachment to both polystyrene and PDMS substrates. Therefore, the culture conditions and design used in this study were suitable for the development of microphysiological systems.

The Young’s modulus of the soft, medium, and hard PDMS used in this study was 4.4, 17, and 26 kPa, respectively [23]. Polystyrene culture plates have higher stiffness at the GPa level [36]. The stiffness of the PDMS substrate regulated the proinflammatory response of the L929 cells. The soft PDMS substrate increased the expression of proinflammatory mediators and proteases, such as *Ptgs2*, *Il1b*, *Il6, Mmp2, and Mmp9*, as well as PGE_2_ production in L929 cells, regardless of ligand stimulation with LPS. However, mid and hard PDMS showed similar expression profiles of the proinflammatory mediators and proteases in L929 cells as the expression on the polystyrene culture plates regardless of the proinflammatory conditions. L929 cells were derived from fibrosarcoma cells. These proinflammatory mediators potentially stimulate osteoclast differentiation of macrophages or osteoclast precursors, leading to inflammatory osteolysis [37,38,39]. In addition, soft PDMS upregulated the *M-csf* expression of L929 cells, whereas hard PDMS downregulated the expression. M-CSF is commonly involved in the differentiation of monocytes into macrophages and in the osteoclast differentiation of monocytes or macrophages together with RANKL [40]. The production of osteoclastogenesis-inducing factors is a signature of the inherent functions of L929 cells [41]. Therefore, the L929 cell supernatant has been used for in vitro osteoclastogenesis [42]. The L929 cell supernatant from the soft PDMS supported osteoclast differentiation of RAW264.7 cells induced by RANKL and TNF-α more effectively than the supernatant from the polystyrene culture plate and the mid and hard PDMS. These observations indicate that the inherent osteoclastogenesis-inducing capability of L929 cells can be regulated by changing substrate stiffness.

Cells sense mechanical cues, such as stiffness or topography, with their mechanosensors, and determine their fate or function when adapting to the microenvironment [43]. The nuclear translocation of YAP in L929 cells was inhibited on the soft PDMS substrate, whereas it was observed on other substrates. Young’s modulus of the soft PDMS used in this study falls within the range of relatively soft skin, whereas the values of the mid and hard PDMS range from muscles to tendons [44]. The same soft PDMS substrate induces increased proinflammatory reactions and reduces ECM production in human gingival fibroblasts via inhibition of YAP nuclear translocation, leading to the activation of a proinflammatory transcription factor, even under normal culture conditions without LPS ligand stimulation [23]. Human gingival fibroblasts are small and rounded, with reduced expression of focal adhesion plaques [23] which are mechanosensors for the surrounding microenvironment [45]. A hard PDMS substrate induces the opposite biological phenomenon in human gingival fibroblasts [23]. Similarly, the regulation of the osteoclastogenesis-inducing ability of L929 cells by PDMS substrate stiffness likely involves the activation of cellular mechanotransduction centered on the YAP-mediated signaling pathway. Notably, the stiffness of soft tissue sarcoma is 2.37 ± 1.49 kPa (range: 0.89–6.3 kPa) [46], which is equivalent to that of the soft PDMS substrate used in this study. These observations indicate that L929 cells sense the optimal mechanical properties of their surrounding microenvironment and maximally exert their innate ability through cellular mechanotransduction, which is a universal phenomenon across species. Notably, the polystyrene cell culture plates were both collagen-coated and culture-graded and were originally indicated for use in cell adhesion experiments. Additionally, the stiffness of soft tissues, such as fat (a few hundred Pa), skin (approximately 60 kPa), and tendons (approximately 1 GPa), was considerably higher than the stiffness range used in this study (4.4, 17, or 26.2 kPa). Furthermore, dynamic mechanical stress exerted by external forces, such as tissue contraction, stretching, and tissue fluid flow, targeting soft tissues, is also involved in cellular responses to the extracellular matrix [47]. This culture study focused only on substrate stiffness, which generates intrinsic mechanical stress (such as static mechanical stress) within cells. Determining the detailed biological mechanisms underlying the mechanoregulation of L929 cells will be of interest to future research.

The *Gm-csf* expression of L929 cells on the soft PDMS was higher than that on the polystyrene culture plate but not significantly different from that on the mid and hard PDMS substrates under normal culture conditions without LPS addition. GM-CSF is known to function as an inhibitory factor for osteoclastogenesis by attenuating the RANK/RANKL signaling pathway or by turning osteoclast precursors back into the macrophage lineage [48,49]. The supernatant of the macrophage cell line polarized into the M1 type by physical stimulation of titanium nanosurfaces with nanospikes inhibited the in vitro osteoclast differentiation of osteoclast precursor cells by producing this inhibitory factor [50]. There was no difference in the in vitro osteoclastogenesis of the L929 supernatant from the soft PDMS substrate compared to those in the conditioned medium with fresh DMEM or the medium without the supernatant. The production of Gm-CSF from L929 cells might alleviate the osteoclastogenesis-inducing effect of the L929 supernatant and be one of the factors that makes it possible to replace exogenous M-CSF application for in vitro osteoclastogenesis [51]. Furthermore, the *Gm-csf* expression of L929 cells on the soft PDMS substrate was low at a level equivalent to that on the polystyrene culture plate under LPS stimulation, in contrast to the much higher expression on the mid and hard PDMS substrates. The *Gm-csf* expression of a macrophage cell line upregulated by physical stimulation of titanium nanosurfaces was further augmented by coexisting LPS ligand stimulation [52]. The proinflammatory reactions of human gingival fibroblasts on soft PDMS substrates are augmented by additional LPS ligand stimulation [23]. Similarly, the synergistic effect of LPS ligand stimulation and modulation of substrate stiffness may more effectively regulate the ability of L929 cells to promote osteoclast differentiation.

Results from our previous study, in which inhibitory reagents were used as mediators of the mechanotransduction signaling pathway, suggested that both YAP-dependent and YAP-independent systems were involved in the regulation of proinflammatory responses of human gingival fibroblasts mediated by PDMS substrate stiffness [23]. Actin cytoskeletal disruption is directly associated with the activation of proinflammatory transcription factors and subsequent cellular proinflammatory responses [53,54]. Here, we observed that L929 cells shrank when cultured in a softer PDMS substrate, which suggests that, in addition to YAP transcription factors, YAP-independent mechanotransduction systems may also be involved in the substrate stiffness-mediated mechanoregulation of L929 cells. In addition, the effects of the self-produced extracellular matrix on substrate stiffness-mediated cellular mechanoregulation remain unknown because only short-term cell culturing periods (up to 3 days) were assayed in this study. However, our previous study suggested that the structure of the self-produced extracellular matrix contributes to the substrate stiffness-mediated cellular mechanoregulatory mechanisms of the extracellular matrix via physical signals [23]. Taken together, these findings demonstrate that the osteoclastogenesis-inducing potential of L929 cells may be controlled by adjusting substrate stiffness, which may pave the way for the development of microphysiological systems. Clarification of the detailed biological mechanisms underlying the substrate stiffness-mediated mechanoregulation of L929 cells using knock-in or knockout experiments or a prolonged culture period may be of interest for future research.

In this study, we showed that substrate stiffness can regulate the osteoclastogenesis-inducing ability of mouse fibroblast cell lines. These cells are well known for their ability to produce osteoclastogenesis-inducing factors and are relatively easy to handle. However, mouse-derived biomolecules do not necessarily act on human cells at the same level as human-derived molecules [55]. Further investigation of the effects of substrate stiffness on the osteoclastogenesis-inducing ability of human-derived cell lines, such as the human myeloid cell line KPB-M15 [56], is needed for the development of microphysiological systems for inflammatory osteolysis. In addition, it is necessary to verify whether the stiffness suitable for controlling a certain cell function is universal for other materials or extracellular matrices used in microphysiological systems. For instance, hydrogel-based materials can provide a space in which cell functions can be tuned synergistically by both the static mechanical stimulation of material stiffness and the ligand stimulation of immobilized biomolecule [57]. Further research interests remain in the future. However, this study successfully showed substrate stiffness-mediated tuning of the osteoclastogenesis-inducing potential of immortalized cell lines, paving the way for spatial cellular control for the development of osteoimmunological microphysiological systems.

## 4. Materials and Methods

### 4.1. Preparation of Stiffness-Controlled Substrates

Commercial PDMS (Sylgard 527; The Dow Chemical Company, Midland, MI, USA) was prepared as a culture substrate according to a previously reported method [23]. A vinyl-terminated base and methyl hydrogen siloxane curing agent were mixed at weight ratios of 5:4, 1:1, and 4:5 to obtain soft (4.4 kPa), mid (17.0 kPa), and hard stiffness (26.2 kPa), respectively. After obtaining the mixture, PDMS substrates were polymerized on the bottom of a tissue culture plate (CELLSTAR^®^, Greiner Bio-One GmbH, Kremsmünster, Austria) and sterilized using a low-temperature plasma sterilizer (RENO-S20, RENOSEM Co., Ltd., Gyeonggi-do, Republic of Korea).

The PDMS substrates were coated with type I collagen for cell attachment. Two types of collagens, bovine dermis-derived native collagen (IAC-30, Koken Co., Ltd., Tokyo, Japan) and atelocollagen (IPC-30, Koken), were used. A polystyrene culture plate or PDMS substrates were coated with each collagen solution at 0.01 wt% and 0.1 wt% concentrations, which were based on the manufacturer’s instructions. The plates were then incubated at room temperature for 90 min.

### 4.2. L929 Fibroblast Cell Cultures

The mouse fibroblast-like cell line L929 (ECACC 85011425, KAC Co., Ltd., Kyoto, Japan) was cultured on collagen-coated PDMS with various stiffness. L929 cells were grown in the L929 growth medium consisting of Dulbecco’s modified Eagle medium/Ham’s F12 (DMEM/F12, Gibco, New York, USA) supplemented with 7% fetal bovine serum (FBS) (Japan Biocealum Co., Ltd., Hiroshima, Japan), 100 U of penicillin, and 100 μg/mL of streptomycin (FUJIFILM Wako Pure Chemical Corporation, Osaka, Japan) at 37 °C in a 5% CO_2_ atmosphere. The cells were then passaged twice. After 80–90% confluences, the cells were detached with 0.25% trypsin/1 mM Ethylene Diamine Tetra-acetic Acid (FUJIFILM Wako Pure Chemical Corporation) and seeded on collagen-coated or non-coated substrates of a 12- or 24-well culture-graded polystyrene plate or soft, mid, or hard PDMS at 1.6 × 10^4^ or 1.0 × 10^5^ cells/cm^2^, respectively. The cells were cultured for up to 3 days at 37 °C in a 5% CO_2_ atmosphere, and the medium was renewed every three days.

The culture supernatants of L929 cells cultured for 12 h on each substrate were collected and filtered through a Millex-Gp 0.22 µm membrane filter (Merck Millipore Ltd., Carrigtwohill, Ireland) into a tube. The supernatants were stored at –80 °C prior to use in the subsequent osteoclast precursor cell experiment.

### 4.3. Culture Conditions for the Induction of a Proinflammatory Response

To evaluate the effect of substrate stiffness on the proinflammatory responses of L929 cells under inflammatory conditions, LPS extracted from *Escherichia coli* O55:B5 cells (Sigma-Aldrich, St. Louis, MO, USA) was used to induce inflammation. The cell cultures were treated with final concentrations of 0, 1, 10, 100, or 1000 ng/mL LPS at cell seeding. The cells were co-incubated with LPS for 12 h at 37 °C in a 5% CO_2_ atmosphere and evaluated for proinflammatory responses.

### 4.4. Mouse Osteoclast Precursor Cell Line Culture

The mouse osteoclast precursor cell line RAW 264.7 (RCB0535) from the Riken BioResource Center (BRC) was co-cultured with the supernatants from the L929 cells cultured on each substrate. The cells were grown in the RAW 264.7 medium consisting of the minimum essential medium and alpha modification (Nacalai Tesque, Kyoto, Japan) supplemented with 10% FBS at 37 °C in a humidified 5% CO_2_ atmosphere. The cells were then passaged twice. At 70% confluency, the cells were detached by scraping and seeded on 24- or 96-well culture-graded polystyrene plates at 7 × 10^4^ cells/mL or 1.125 × 10^4^ cells/mL in the RAW 264.7 medium. According to a previously reported protocol, 50 ng/mL recombinant human soluble RANKL (Oriental Yeast, Tokyo, Japan) and 50 ng/mL TNF-α (aa80-235, R&D System, Minneapolis, MN, USA) were added to the RAW 264.7 culture medium for osteoclast differentiation. Fresh DMEM/F12 basal medium or the L929 culture supernatants were mixed with RAW 264.7 medium in a 1:9 volume ratio for 5 days and then evaluated for TRAP staining, osteoclast cell number, and osteoclast-related marker gene expression.

### 4.5. Reverse Transcription–Polymerase Chain Reaction (RT–PCR)

Total RNA in the culture was extracted using TRIzol reagent (Ambion/Life Technologies, Carlsbad, CA, USA) on PDMS and polystyrene plates. RNA was isolated and purified using the RNAeasy^®^ Mini Kit (Qiagen, Hilden, Germany), followed by DNase treatment and removal (Thermo Fisher Scientific, Waltham, MA, USA). Complementary DNA (cDNA) was synthesized using a PrimeScriptTM II 1st Strand cDNA Synthesis Kit (Takara Bio, Shiga, Japan). Messenger RNA (mRNA) expression was determined using agarose gel electrophoresis or real-time RT–PCR. For agarose gel electrophoresis, PCR was performed using Taq DNA polymerase (Go Taq; Promega Corporation, Madison, WI, USA). The primers are listed in Supplementary Table S1. The PCR products were visualized on a 1.5% agarose gel using ethidium bromide staining. The band intensity was detected under UV light and normalized to glyceraldehyde 3-phosphate dehydrogenase (Gapdh) mRNA. For real-time PCR, mRNA expression was determined using the StepOnePlus Real-Time PCR system (Thermo Fisher Scientific) and the TaqMan™ Fast Advanced Master Mix (Thermo Fisher Scientific) or Thunderbird™ SYBR™ qPCR Mix (Toyobo, Osaka, Japan) for TaqMan probe-and SYBR green-based PCR reactions, respectively. Target gene expression levels were quantitatively analyzed using the comparative cycle time (ΔΔCT) method. Gapdh was used as the housekeeping gene. The primers used are listed in Supplementary Tables S2 and S3.

### 4.6. Cell Viability and Attachment Assays

Methylene blue staining was used to visualize the attached L929 cells on the collagen-coated polystyrene culture plate and the PDMS 12 h after seeding. The cells were fixed with 10% buffered formalin (FUJIFILM Wako Pure Chemical Corporation) and stained with 1.4% methylene blue solution (Sigma-Aldrich) for 30 min at room temperature. After washing with 10 mM sodium borate buffer (Sigma-Aldrich), stained cells were observed under a light microscope.

The cell viability on collagen-coated polystyrene culture plates and PDMS 12 h after seeding was quantified using a tetrazolium salt-based assay (WST-1; Roche Diagnostics, Tokyo, Japan). For WST-1-based colorimetry, 10% (*v*/*v*) WST-1 reagent was added to the culture medium. The culture plate was incubated at 37 °C for 3 h, and then the supernatants were transferred into a 96-well microplate. The amount of formazan produced in the supernatant was measured using an ELISA plate reader at 450 nm.

### 4.7. Cell Morphometry Analysis

The cells were observed under a phase microscope. Cell morphometries for cell area, perimeter, and Feret diameter were performed on microscope phase images using ImageJ software version 1.53t (National Institutes of Health, Bethesda, MD, USA). Feret diameter was defined as the length of the longest straight line connecting any two points on the outer boundary of the selection, as determined using ImageJ with default settings.

### 4.8. Quantification of Prostaglandin E2

The PGE_2_ concentration in the culture supernatant was quantified using a highly sensitive competitive immune assay (ADI-900-001; Enzo Life Sciences, Farmingdale, NY, USA). The assay is based on a competitive reaction to an anti-PGE_2_ monoclonal antibody between alkaline phosphatase covalently bound to PGE_2_ and the sample. Briefly, samples or PGE_2_ standards were co-incubated with an anti-PGE_2_ monoclonal antibody and alkaline phosphatase covalently bound to PGE_2_ in a 96-well G × M IgG microtiter plate for 2 h at room temperature on a shaker. After washing with the unbound antibody and PGE_2_, the plates were incubated with p-nitrophenylphosphate for 45 min at room temperature to develop a color against alkaline phosphatase. The optical density of the plates was measured at 405 nm using a microplate reader. The PGE_2_ concentration was quantified against a standard curve.

After collecting the culture supernatants for PGE_2_ quantification, the total DNA in the adherent cells was measured using a DNA quantification kit (COSMO BIO Co., Ltd., Tokyo, Japan) according to the manufacturer’s instructions. After washing with phosphate-buffered saline (PBS), the cells were lysed with −80 °C freezing and thawing cycles in a cell lysis buffer. The cell lysates were mixed with an equal volume of Hoechst 33258 and diluted 20-fold using the same cell lysis buffer. The mixture was transferred to 96-well black flat-bottom microplates. Fluorescence intensity was measured using a GloMax-Multi Detection System reader (Promega Corporation, Madison, WI, USA) (excitation at 365 nm and emission: 410 at 460 nm).

The concentration of PGE_2_ was normalized to the DNA concentration of adherent cells on each substrate and expressed as the PGE_2_ level per unit of DNA in adherent cells.

### 4.9. Immunofluorescence Staining and Analyses

The cells on the collagen-coated polystyrene culture plate and PDMS were fixed using 4% paraformaldehyde phosphate buffer solution (FUJIFILM Wako Pure Chemical Corporation) for 15 min. After washing with PBS, the cells were blocked for non-specific protein binding using a blocking buffer containing 2.0% bovine serum albumin (BSA) (FUJIFILM Wako Pure Chemical Corporation), 0.1% Triton-X (FUJIFILM Wako Pure Chemical Corporation), and 0.01% Tween 20 (Sigma-Aldrich) for 60 min. The cells were then incubated in a mixed solution consisting of 1/500 rhodamine phalloidin (Thermo Fisher Scientific) for *F*-actin staining and 1/500 Hoechst 33258 pentahydrate (bis-benzidamine) (Thermo Fisher Scientific) for nuclear staining. In the cases to detect the following specific cellular marker, the cells were fixed, permeabilized, and blocked for non-specific protein and incubated with a primary antibody, such as 1/200 anti-YAP (sc-101199, Santa Cruz Biotechnology, Dallas, TX, USA), overnight at 4 °C. After washing with PBS, the cells were incubated with secondary antibodies Alexa Fluor 488 (H&L), 1/500 Hoechst 33258 pentahydrate, or 1/500 rhodamine phalloidin for 90 min at room temperature in the dark. The cells were washed with PBS and mounted on a glass-bottomed dish (Matsunami Glass Ind., Ltd., Osaka, Japan) containing 90% glycerol. The cells were observed using an LSM 780 confocal laser microscope (Carl Zeiss, Jena, Germany). To determine the percentage of cells with nuclear YAP, the number of cells with and without nuclear YAP was counted in randomly selected low-magnification images using ImageJ software version 1.53t.

### 4.10. TRAP Staining

After co-culturing with or without RANKL and TNF-α and culture supernatants from L929 cells at 12 h, RAW264.7 cells cultured on polystyrene surfaces were fixed with 10% neutral buffer formalin on ice for 10 min, washed with PBS, and incubated in an ethanol/acetone (50:50 *v*/*v*) mixture at −30 °C for 1 min. The cells were again washed with PBS and incubated in a mixture solution of tartaric acid and acid phosphatase substrate (FUJIFILM Wako Pure Chemical Corporation) at 37 °C for 30 min. TRAP-positive cells with more than three nuclei were defined as osteoclasts under an optical microscope. The number of osteoclasts and the percentage of TRAP-positive area relative to the total culture growth area were measured on microscopic images using ImageJ software version 1.53t.

### 4.11. Statistical Analysis

All culture experiments, except for agarose gel electrophoresis-based RT–PCR analysis and immunofluorescence staining, were performed on at least three independent cell batches on multiple days (*n* = 3). Cell morphometries of cell shapes were analyzed in single cells using randomly selected phase-contrast microscopic images from multiple cultures (*n* = 15). The number of osteoclast cells, TRAP-positive areas, and percentage of cells with nuclear YAP were analyzed in four randomly selected phase-contrast or confocal microscopic images in multiple cultures (*n* = 4). One-way analysis of variance (ANOVA) or the Kruskal–Wallis H-test was used to assess differences among multiple experimental groups, whereas a two-way ANOVA was used to assess the interactions between differences in substrate types and medium components. When appropriate, a post hoc Tukey’s honestly significant difference test or the Dann–Bonferroni test was performed. A *p*-value of <0.05 was considered statistically significant. All statistical analyses were performed using IBM SPSS Statistics version 21 (IBM Japan, Ltd., Tokyo, Japan).

## 5. Conclusions

PDMS-based substrate stiffness tunes osteoclastogenesis-inducing potentials of the mouse fibrosarcoma cell line by controlling YAP-mediated cellular mechanotransduction. Soft substrates mimicking the stiffness of biological soft tissues can enhance the osteoclas-togenesis-inducing potential of L929 cells by inhibiting the nuclear translocation of YAP, whereas harder substrates have minimal effects on the L929 cellular response.

## Data Availability

All raw and processed data in the present study are available from the corresponding author on reasonable request.

## Supplementary Materials

The following supporting information can be downloaded at: https://www.mdpi.com/article/10.3390/ijms24108959/s1.
