# Supplementary material for: Mechanoregulation of Osteoclastogenesis-Inducing Potentials of Fibrosarcoma Cell Line by Substrate Stiffness
Source: Int J Mol Sci. 2023 May 18;24(10):8959. doi: 10.3390/ijms24108959 (PMC10219368; doi:10.3390/ijms24108959)
Supplement: Supplementary file 1 [file ijms-24-08959-s001.zip › ijms-2362084-supplementary.pdf]

Table S1. A primer list of mouse genes for the agarose gel electrophoresis- based PCR reactions

Encoded protein name (gene name)	Primers (Fw, forward; Rv, reverse)	Product size (bp)	Accession number
**Mouse**			
Prostaglandin G/H synthase 2 *(Ptgs2)*	Fw: 5'-CAGTCAGGACTCTGCTCACG-3'<br>Rv: 5'-TTGACATGGATTGGAACAGC-3'	171	NM_011198.4
Interleukin-6 *(Il6)*	Fw: 5'-CACTTCACAAGTCGGAGGCTTA-3'<br>Rv: 5'-GCAAGTGCATCATCGTTGTTC-3'	111	NM_031168.2
Glyceraldehyde 3 Phosphate Dehydrogenase *(Gapdh)*	Fw: 5'-CACCATGGAGAAGGCCGGGG-3'<br>Rv: 5'-GACGGACACATTGGGGGTAG-3'	418	NM_001289726.1

Table S2. A primer list of mouse genes for the SYBR-green-based PCR reactions

Encoded protein name (gene name)	Primers (Fw, forward; Rv, reverse)	Product size (bp)	Accession number
**Mouse**			
Prostaglandin G/H synthase 2 *(Ptgs2)*	Fw: 5'-CAGTCAGGACTCTGCTCACG-3'<br>Rv: 5'-TTGACATGGATTGGAACAGC-3'	171	NM_011198.4
Interleukin-1β *(Il1b)*	Fw: 5'-AAGGGCTGCTTCCAAACCTTTGAC-3'<br>Rv: 5'-ATACTGCCTGCCTGAAGCTCTTGT-3'	100	NM_008361.4
Interleukin-6 *(Il6)*	Fw: 5'-CACTTCACAAGTCGGAGGCTTA-3'<br>Rv: 5'-GCAAGTGCATCATCGTTGTTC-3'	111	NM_031168.2
Matrix Metallopeptidase-2 *(Mmp2)*	Fw: 5'-GATAACCTGGATGCCGTCGTG-3'<br>Rv: 5'-CTTCACGCTCTTGAGACTTTGGTTC-3'	146	NM_008610.3
Matrix Metallopeptidase-9 (MMP-9) *(Mmp9)*	Fw: 5'-GCCCTGGAACTCACACGACA-3'<br>Rv: 5'-TTGGAAACTCACACGCCAGAAG-3'	85	NM_013599.4
Macrophage Colony Stimulating Factor *(M-csf)*	Fw: 5'-TACAAGTGGAAGTGGAGGAGCCAT-3'<br>Rv: 5'-AGTCCTGTGTGCCCAGCATAGAAT-3'	146	NM_007778.4
Granulocyte Macrophage Colony-Stimulating Factor *(Gm-csf)*	Fw: 5'-TGGGCATTGTGGTCTACAGC-3'<br>Rv: 5'-GCGGGTCTGCACACATGTTA-3'	191	NM_009969.4
Glyceraldehyde 3 Phosphate Dehydrogenase *(Gapdh)*	Fw: 5'-TGCACCACCAACTGCTTAG-3'<br>Rv: 5'-GGATGCAGGGATGATGTTC-3'	177	NM_001289726.1

Table S3. A primer list of mouse genes for the TaqMan probe-based PCR reactions

Encoded protein name (gene name)	Accession number
**Mouse**	
Acid Phosphatase 5, Tartrate Resistant *(Acp5)*	NM00475698_m1
Cathepsin K *(Ctsk)*	NM00484039_m1
